# Contactless Inductive Bubble Detection in a Liquid Metal Flow

**DOI:** 10.3390/s16010063

**Published:** 2016-01-06

**Authors:** Thomas Gundrum, Philipp Büttner, Bachir Dekdouk, Anthony Peyton, Thomas Wondrak, Vladimir Galindo, Sven Eckert

**Affiliations:** 1 Institute of Fluid Dynamics, Helmholtz-Zentrum Dresden-Rossendorf, Bautzner Landstr. 400, Dresden 01328, Germany; buettnerphilipp@gmx.de (P.B.); t.wondrak@hzdr.de (T.W.); v.galindo@hzdr.de (V.G.); s.eckert@hzdr.de (S.E.); 2 School of Electrical and Electronic Engineering, The University of Manchester, Manchester M13 9PL, UK; bachiruk@hotmail.com (B.D.); a.peyton@manchester.ac.uk (A.P.)

**Keywords:** contactless inductive measurements, two phase flow, liquid metal, bubble detection, void fraction

## Abstract

The detection of bubbles in liquid metals is important for many technical applications. The opaqueness and the high temperature of liquid metals set high demands on the measurement system. The high electrical conductivity of the liquid metal can be exploited for contactless methods based on electromagnetic induction. We will present a measurement system which consists of one excitation coil and a pickup coil system on the opposite sides of the pipe. With this sensor we were able to detect bubbles in a sodium flow inside a stainless steel pipe and bubbles in a column filled with a liquid Gallium alloy.

## 1. Introduction

Bubble detection and void fraction measurement is of huge interest in two-phase flow research and in industrial applications like continuous casting of steel [1], liquid metal cooled reactors or cracking of Methane and Ethane into hydrogen inside a liquid metal reactor [2]. For example, in continuous casting argon gas is injected into the submerged entry nozzle (SEN) in order to reduce nozzle clogging and to withdraw impurities from the melt [1]. Important parameters include the filling level of the SEN and the bubble distribution in the melt. Both factors have a strong influence on the flow structure in the mold and therefore on the quality of the produced steel. Due to the opaqueness and the high temperature of the liquid steel, only a limited number of measurement systems is available. One of them is the Mutual Inductance Tomography (MIT) which is able to reconstruct the electrical conductivity distribution in a cross section of a pipe. It was already used to visualize the position of the liquid metal strand in the submerged entry nozzle in a real caster [3] and in a cold model [4,5]. In case of the cold model experiments the gas distribution in a liquid metal two-phase flow in a pipe is reconstructed by solving a non-linear inverse problem for magnetic field data recorded by 8 sensors. In order to reduce the complexity of this system for detecting bubbles in a pipe filled with liquid metal, we developed a new system consisting only of one excitation coil and one planar gradiometer coil on the opposite sides of the pipe [6]. The planar gradiometer consists of two pickup coils positioned on top of each other which are wound in opposite direction and are connected in series. In this setup they are only sensitive to any asymmetric magnetic field distribution with respect of the two receiver coils over the height of the pipe. This enhances the signal to noise ratio between the symmetric magnetic field generated by the excitation coil and the distortion of the magnetic field induced by the bubble [7].

The primary objective of this sensor is the detection of bubbles which are floating in the liquid metal and traversing the sensitive region of the sensor. This could be important for the leakage detection in sodium/water heat exchangers. If the liquid sodium comes in contact with water or steam the oxygen of the water molecules reacts with the sodium and the hydrogen remains as gas phase in the liquid sodium [8,9]. For instance, if the sensor is located downstream the heat exchanger, then the detection of gas bubbles in the sodium will indicate a leakage of water in the heat exchanger.

In this paper we will briefly describe the working principle of the sensor and present first measurements in the eutectic alloy GaInSn and in liquid sodium.

## 2. Measuring Principle

In contrast to the well established electromagnetic identification of conducting objects in a non-conducting volume [10] the task to reconstruct an argon bubble inside the liquid metal is similar to the case of the detection of cracks using non-destructive eddy current testing [11]. Instead of moving the sensor along the object containing the cracks, in our case the bubble detector is fixed on the pipe and the bubble moves through the detector. The basic idea of this sensor is that the bubble in the liquid metal acts as an insulating obstacle to the eddy currents generated by the magnetic field of the excitation coil. The slight change of the current distribution can be detected outside the liquid metal by a magnetic field sensor. The challenge of this method is the reliable detection of these small variations which requires a sensitive measurement system.

**Figure 1:**
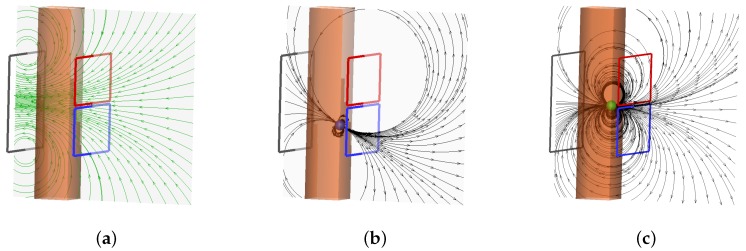
Principle of the measurement system with the magnetic field structure at the central plane of the excitation coil and its deformation due to the presence of a bubble. (**a**) The field lines of the magnetic field generated by the excitation coil; (**b**) Field deformation due to the presence of a bubble in lower section; (**c**) Field deformation due to the presence of a bubble at the equatorial plane. The induced voltages in both induction coils of the planar gradiometer have the same amplitude but opposite sign and therefore they cancel each other out.

Figure 1a shows a schematic sketch of the sensor. On the left hand side of the pipe the gray colored excitation coil is located. The excitation coil generates the primary magnetic field for the measurement indicated by the green colored field lines at the central plane. For illustrative purpose we created a simple 2D-model in which the magnetic field of the excitation coil is calculated using two horizontal infinite long conductors with opposite current directions. They represent the upper and lower horizontal wires of the rectangular transmitting coil. The distance between the conductors is 135 mm which is the same distance of the wires of the excitation coil in the experiment.

On the opposite side, the planar gradiometer is mounted symmetrically with respect to the excitation coil. The planar gradiometer is composed of two coils, colored red and blue with opposite turning direction. The advantage of this arrangement is that the planar gradiometer neglects the primary magnetic field generated by the excitation coil. If there is no bubble in the measurement volume, the output signal of the planar gradiometer is zero, because the induced voltages in both parts are equal, and due to the opposite winding directions of the induction coils they cancel each other out. If a bubble is entering the measurement volume from below, as depicted in Figure 1b, the eddy currents in the liquid metal are deformed and the change of the so-called secondary magnetic field is visualized by the black field lines. In order to account for this deformation in the 2D model the secondary magnetic field is modeled by a magnetic dipole with a distance of 6 mm from the infinite long conductors. The dipole is aligned to the excitation field in Figure 1a due to Lenz’s law. The deformation of the field lines results in a non-symmetrical magnetic field distribution in the gradiometer which generates a net voltage signal at the terminals. If the bubble reaches the equatorial plane the induced voltage becomes zero again, because the secondary magnetic field is symmetric with respect of the gradiometer, as depicted in Figure 1c. When the bubbles moves up further, again a voltage signal in the gradiometric coil is generated, but now with an opposite sign.

**Figure 2:**
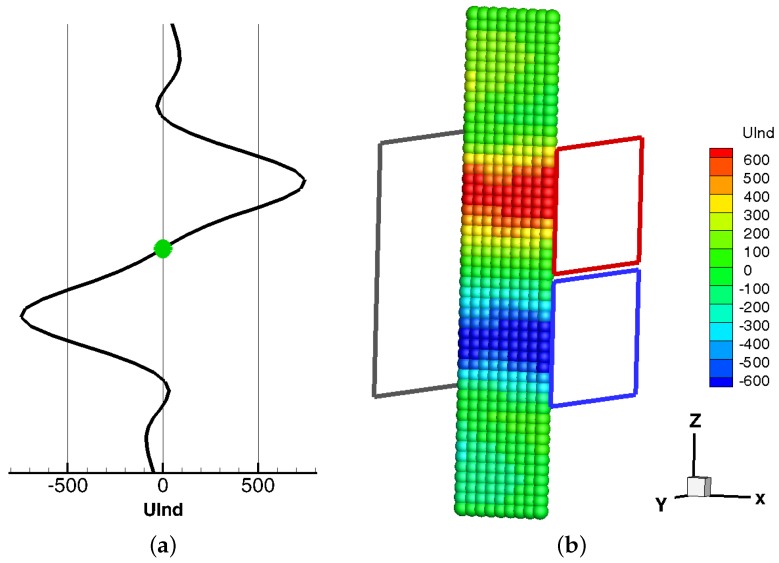
Induced voltage (UInd) of the 2D-model in arbitrary units for a bubble in the channel traveling over z. (**a**) The signal of the gradiometer for one bubble at the center of the channel; (**b**) The color of each bubble in the central plane of the sensor represents its induced voltage in the gradiometer.

In summary, for a bubble traversing the sensor from below, the typical time-dependent voltage signal with a “S”—Shape as depicted in Figure 2a appears. From this signal the vertical position of the bubble can be deduced. One important feature of this setup is the zero crossing of the induced voltage exactly at the time when the bubble passes the equatorial plane of the gradiometer, as marked by the green sphere in Figure 2a. This allows the automatic detection of bubbles without any calibration. Additionally, the rising velocity of the bubbles can be inferred by taking into account the time delay from the minimum to the maximum of the signal at Figure 2a. It is interesting to note that the two zero crossings of the induced voltage calculated by the simple 2D-model in the upper and lower region of the sensor region could also be observed in the measurements. In order to compare the induced voltages for different bubble positions, Figure 2b shows bubbles at various positions in the central plane. The colors of the bubbles indicate the voltage induced in the gradiometric coil for each bubble. In contrast to the strength of the primary field generated by the excitation coil in Figure 2a the secondary field is several orders of magnitude lower.

## 3. Measurement Setup

**Figure 3:**
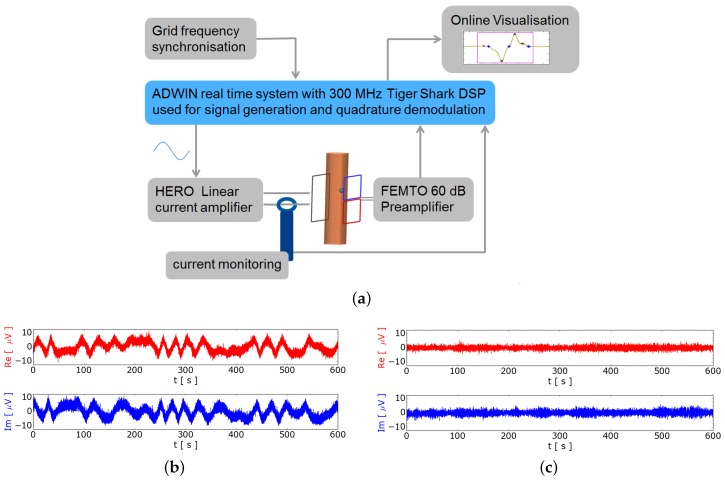
Measurement setup with synchronization to the grid (**a**) Signal generation and instrumentation; (**b**) Free running excitation; (**c**) Excitation synchronized to the grid.

Figure 3a shows the principle setup of the measurement system. The main parts are the excitation coil and the planar gradiometer mounted on opposite sides of the pipe. The core of the measurement system is the real time system (RTS) ADWIN-Pro (Jäger Computergesteuerte Messtechnik GmbH) with a processor clock of 300 MHz. The sinusoidal waveform with a typical frequency of 100 Hz for the excitation current is generated with a 16 bit DA-converter of the RTS. To get a minimal phase shift between the generated voltage and the current, a current amplifier with 10 kHz bandwidth is used for driving the excitation coil. The current amplifier generates a proportional output current to the input voltage supplied from the DA-converter. The excitation current is measured by a DC to 100 MHz current clamp which is connected to a 18 bit AD-converter of the RTS. The measurement signal from the planar gradiometer is amplified with a voltage amplifier by typically 60 dB gain and recorded with a 18 bit AD-converter in the RTS. Inside the RTS, phase sensitive quadrature demodulation is carried out and streamed through a FIFO and the TCP/IP network for online visualization or post processing. The data synthesis of the excitation signal and the data acquisition are strictly coupled to achieve an optimal sensitivity. The data rate for the AD- and DA-converters is initialized to approximately 19.2 kHz. To reduce the influence of the electromagnetic interference (EMI) from the power supply grid the data rate is coupled to the power supply grid. This coupling modifies slightly the data rate of 19.2 kHz. A bubble free zero measurement without synchronization to the grid is shown at Figure 3b. In contrast Figure 3c shows a zero measurement using digital phase looked loop (PLL) for grid synchronization. This synchronization reduces the EMI by a factor of 5, which is determined by the quotient of the standard deviations from Figure 3c to Figure 3b. This shows that a RTS is needed to achieve a stable synchronized data synthesis and data acquisition with the power net using a programmed PLL.

With this configuration a simple online visualization of bubbles is possible. Using additional signal processing described in Section 4.1, the signal of the bubbles can be enhanced and processed further. The plotted voltages represent always the induced voltages at the gradiometer fed to the pre-amplifier and not the acquired voltage at the AD-converter.

We injected argon with an electronic mass flow controller (MFC). The flow rates presented in this paper are in standard cubic centimeter per minute (sccm) of argon at normal pressure.

## 4. Bubble Detection within Liquid GaInSn

**Figure 4:**
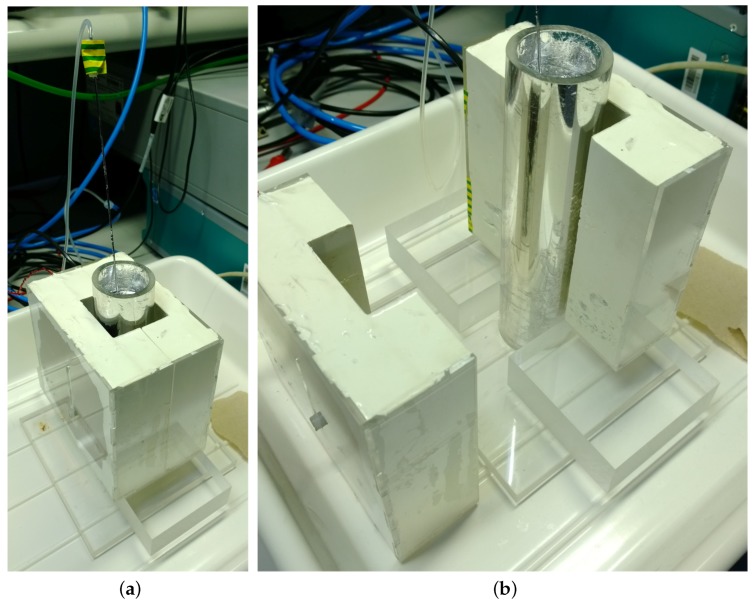
Photograph of the cylindrical setup with the vessel filled with GaInSn, the excitation coil and the planar gradiometer. (**a**) Overview of the setup; (**b**) Detailed view by removing the excitation coil.

We set up a cylindrical vessel with an inner diameter of 40 mm filled with eutectic GaInSn, which is liquid at room temperature. Figure 4 shows a photograph of the setup with the bubble sensor. Argon gas was injected into GaInSn through a thin stainless steel tube with an inner diameter of about 1 mm which produced bubbles with a diameter of approximately 5 mm. The signal processing is described in the following section.

In order to estimate the possible influence of the electromagnetically driven fluid flow in the pipe on the bubble flow, numerical simulations were performed. The distributions of the electromagnetic field and the induced Lorentz force density in the pipe were calculated using the finite element code OPERA 3D from Cobham plc. The amplitude of the magnetic field in the liquid metal is of the order of 1.6 mT. Furthermore, the induced flow in the pipe was simulated using the finite volume library OpenFOAM. As a result, velocities in the order of 1 mm/s were obtained in the region near the excitation coil. For the bubble detection and flow rate estimation this effect can be considered as negligible. More details concerning this issue will be published elsewhere.

### 4.1. Signal Processing

**Figure 5:**
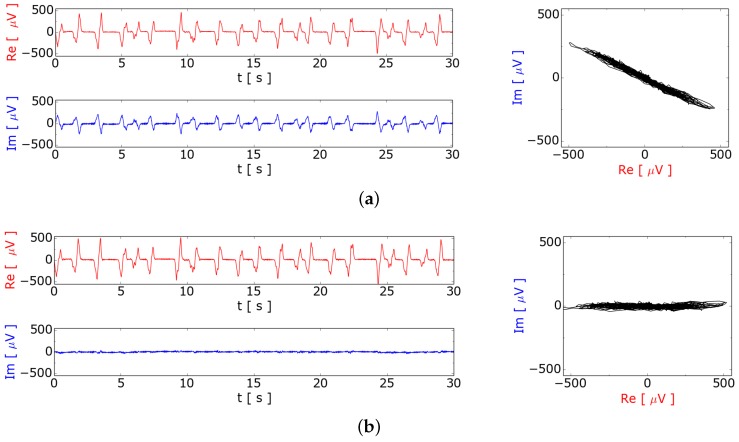
Processing of the quadrature demodulated signal from the planar gradiometer. The real part (I) is red and the imaginary part (Q) is blue in the plots. (**a**) Measured induced voltage ReA and ImA of the measurement system after quadrature demodulation and subtracting the average; (**b**) Signal rotated in the complex plane in order to minimize the imaginary part and maximize the real part.

The data stream of the RTS contains the in phase (I) real part and the quadrature (Q) imaginary part of the demodulated data. Figure 5a shows the acquired time-dependent signal of the red colored real part ReA and the blue colored complex part ImA of the signal. To suppress the influence of a signal from the velocity and from a displacement between the transmitting coil and the planar gradiometer we subtracted the average before. Both signals are evidently correlated, which can be seen in the plot in the complex plane on the right hand side. To simplify the data analysis the signal can be rotated in the complex plane to increase the real part and reject the imaginary part of the signal. For calculating the phase angle ϕ of the signal in the complex plane the linear regression between ReA and ImA is used. The signal is transformed by rotation in the complex plane using following equation:

(1) Re=ReAcos(ϕ)+ImAsin(ϕ)Im=ImAcos(ϕ)-ReAsin(ϕ)

Figure 5b shows the induced signal rotated in the complex plane and the time dependence of the real and imaginary part at a flow rate of 5.6 sccm argon. In the remaining paper, only the rotated real part as ${\text{U}}_{\text{Ind}}$ is used.

### 4.2. Automatic Bubble Detection

**Figure 6:**
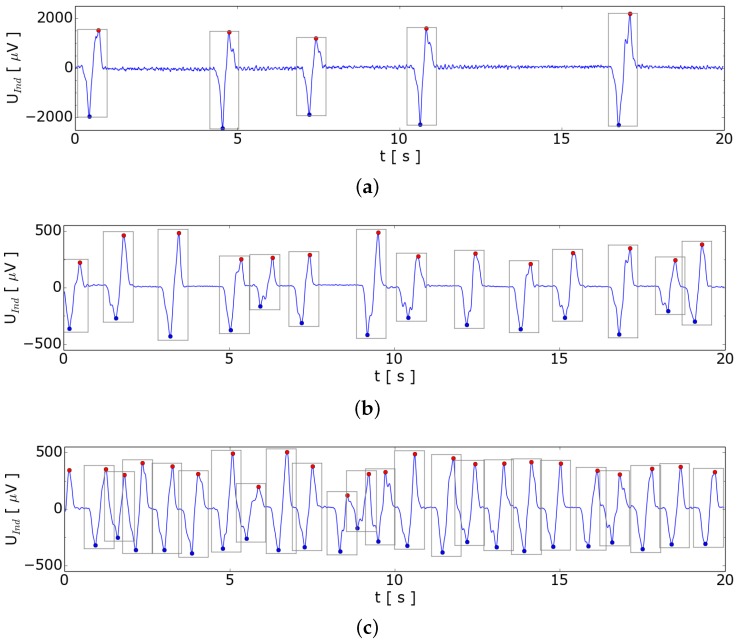
Demodulated signal from argon bubbles rising in the GaInSn column with automatic bubble detection. The bubbles are marked by rectangles; (**a**) Induced signal at 5.2 sccm argon with an average bubble diameter of 8.7 mm; (**b**) Induced signal at 5.6 sccm argon with an average bubble diameter of 6.3 mm; (**c**) Induced signal at 6.3 sccm argon with an average bubble diameter of 5.6 mm.

In the first experiments we generated single bubbles and recorded the induced voltages of the gradiometer. The results of this measurement are presented in Figure 6. The typical shape of the signal for each bubble agrees with the signal predicted by the 2D-model as shown in Figure 2a. The flow rate in this experiment rose from 5.2 to 6.3 sccm. The bubble volume could be estimated taking the volume of gas injected by the MFC divided by the number of bubbles during this period. Afterwards the diameter can be calculated using an spherical model of the bubble. For the bubble diameter calculation in GaInSn normal pressure is used and the hydrostatic pressure is neglected.

Comparing the estimated bubble sizes with the amplitude of the induced voltage in Figure 6 for the three different gas flow rates, it is evident that the induced voltage increases with the bubble size. This indicates that the bubble size could probably be estimated by the amplitude. But one has to be aware of that changing the position of the bubble in x-direction has a similar effect on the amplitude as seen in Figure 2b. It should be analyzed in future how these two information could be extracted from the measurement or if more sensing coils are needed.

For the measurements presented in Figure 6 we observed that the bubbles leave the liquid metal surface at the center of the vessel. Therefore, we assume that the bubbles move along a direct path from the injection nozzle to the top. In this case the amplitude can be correlated with the bubble size. Looking at the measurement in Figure 6a we observe different bubble sizes over time. It seems that the bubble size increases with the time delay after the previous bubble. A reason for the different bubble sizes could be some combined effect of wetting of the inlet and of surface tension.

For this setup, an algorithm for the detection of bubbles was implemented. This algorithm detects the local extremes and the zero crossings of the signal and checks whether the sequence of the events matches the typical sequence for a single bubble. The automatically detected bubble is marked in Figure 6 by a rectangle which visualizes the bubble. This automatic algorithm is expected to function as a robust and useful tool for leakage detection to improve the safety of sodium to water heat exchangers.

### 4.3. Towards Void Fraction Measurement

**Figure 7:**
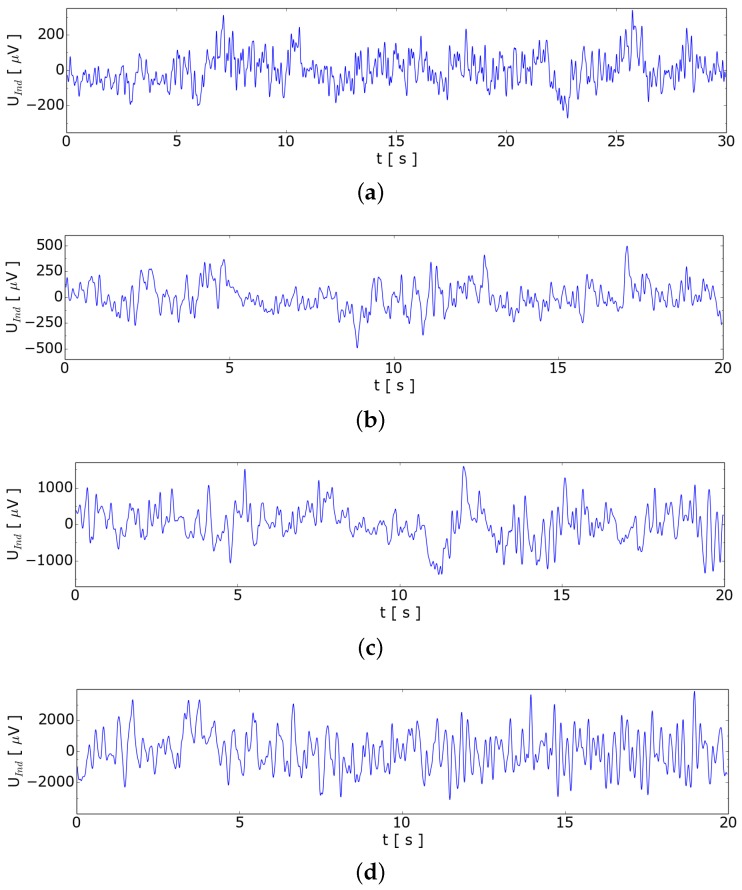
Induced voltage for void fraction analysis. (**a**) 14 sccm—SDEV = 93 μV; (**b**) 22 sccm—SDEV = 154 μV; (**c**) 56 sccm—SDEV = 516 μV; (**d**) 97 sccm—SDEV = 1336 μV.

**Figure 8:**
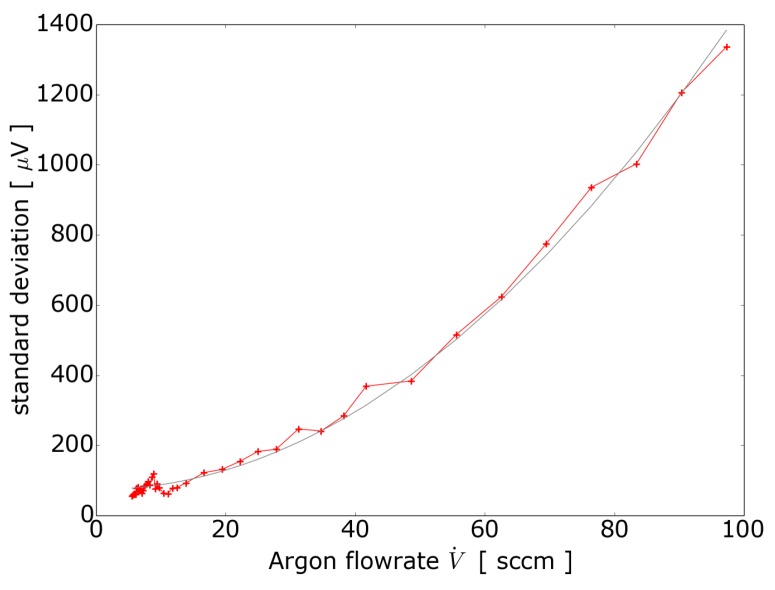
Quadratic dependence of the standard deviation of the induced voltage with the argon flow rate. The red line are the measurements and the grey line is a quadratic fit to the measurement data.

In order to investigate how the measurement signal depends on higher gas flow rates, we carried out measurements where single bubbles are not clearly identified in the signal. To find out the behavior for several bubbles in the measuring volume, we increased the argon flow rate in Figure 7 up to 97 sccm. It turned out that this sensor is able to detect single bubbles up to an argon flow rate of 6 sccm corresponding to approximately 1 bubble per second. For higher flow rates we observed a quadratic dependence of the standard deviation on the flow rate of the argon which is shown in Figure 8.

## 5. Bubble Detection within Liquid Sodium

**Figure 9:**
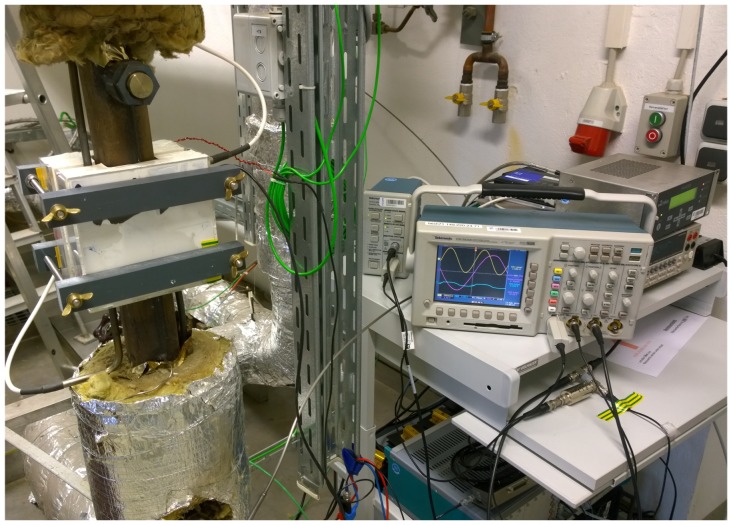
Installation of the sensor at the sodium loop.

In order to evaluate this new measurement system at higher temperatures, we mounted the sensor on a vertical pipe with dimension of 50 mm × 45 mm at the NATAN [12] sodium loop at the Helmholtz-Zentrum Dresden-Rossendorf (HZDR). Figure 9 shows a photograph of the sensor in our sodium laboratory.

**Figure 10:**
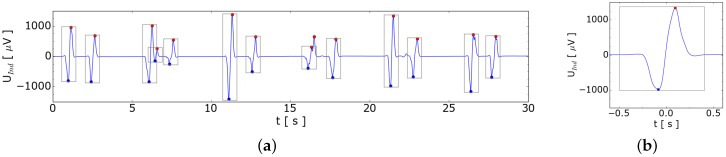

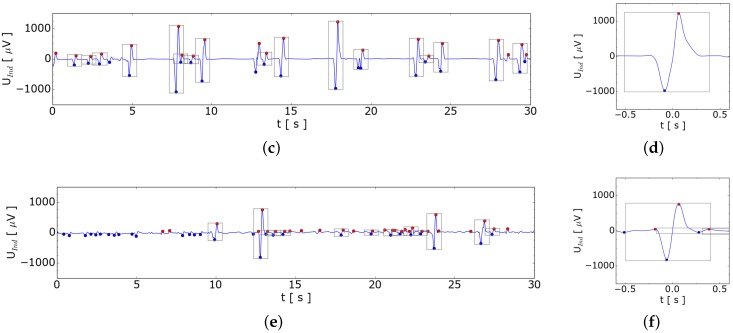
Bubble detection in sodium at rest and in motion, driven by an electromagnetic (EM)-pump. (**a**,**b**) Measured voltage when sodium is at rest with zoom at *t* = 31.4 s; (**c**,**d**) Measured voltage for sodium velocity of 6 cm/s with zoom at *t* = 17.8 s; (**e**,**f**) Measured voltage for sodium velocity of 14 cm/s with zoom at *t* = 12.8 s.

Similar to the experiments with GaInSn we injected 7 sccm argon into the sodium below the sensor. The system pressure in the sodium loop was approximately 1 bar, which likely reduces the bubble size. For bubble diameter calculation we assumed the double pressure in the bubble compared to the GaInSn experiments. To make the experiment more representative with respect to an industrial sodium cooling loop, we switched on the electromagnetic pump generating a maximum sodium velocity of 14 cm/s. Figure 10 shows the results of those experiments. At maximum sodium speed, a current of 400 mA with 50 Hz in the liquid sodium in stream-wise direction was measured which is generated by the EM-pump. This current could be the reason, that the induced bubble signal decreases with increasing fluid velocity. Even in such a noisy environment the characteristic signal of a bubble can be measured. In former experiments without the synchronization to the grid such measurements failed. Compared to the measurements presented at Figure 3b the EMI due to the EM-pump at the sodium loop is several orders of magnitude higher.

## 6. Bubble Velocity Estimation within Liquid Sodium

The automatically detected bubble is tagged in Figure 10 by a rectangle. The minimum is marked with the blue and the maximum with a red point. The time difference between the extremes could be used for the velocity estimation. Assuming that the extremes are in the center of the two coils of the planar gradiometer the velocity could be inferred. Taking the distance of 72 mm between the two coils of the planar gradiometer as a characteristic length for the velocity computation is a critical point and should be analyzed in the future. The estimated bubble velocity is in a reasonable agreement with the Mendelson equation [13] assuming a bubble radius of *r* = 3 mm

(2) $$v_{B}=\sqrt{\frac{\sigma }{r\rho }+g\;r}=0.3\;\frac{m}{s}\;$$

The bubble radius was computed from the argon flow rate and from the number of bubbles per time interval at 1 bar system pressure. For liquid sodium the surface tension *σ* = 0.19 N/m and the density *ρ* = 927 kg/m${}^{3}$ are used. Gravitation acceleration *g* is 9.81 m/s${}^{2}$. Table 1 presents the velocities of the bubbles and of the liquid observed in the sodium experiment.

**Table 1:** Sodium and estimated bubble velocity.

Current of the EM-Pump	0	10	20	(A)
Sodium Velocity	0	6	14	(cm/s)
Bubble Velocity	36	51	61	(cm/s)

## 7. Conclusions

In this paper, we have demonstrated the applicability of a simple contactless inductive sensor consisting of an excitation coil and a planar gradiometer for detecting single bubbles in a pipe filled with liquid metal. In GaInSn as well as in sodium single bubbles can be clearly identified by the voltage measurements. Using a planar gradiometer allows for a robust automatic detection of bubbles without any calibration, since the zero crossing of the induced voltage provides an exact time when the bubble is in the middle of the gradiometer. We have implemented an algorithm for detecting the bubbles automatically. From these data even the rising velocity of the bubble can be estimated. For a more detailed validation of the new inductive method, accompanying ultrasound measurements are under preparation. Additionally, we will investigate if it is possible to infer the bubble position in the cross section of the channel from measurements using a more sophisticated configuration.

There are first indications that the ratio of imaginary to real part of the signal at Figure 5a gives some information about the bubble size versus conductivity of the liquid metal, which should be analyzed in the future. The reason for this assumption is, that the bubble itself reduces the ohmic losses due to eddy currents and changes the mutual inductance itself. Both effects are perpendicular in the complex plane and can influence the ratio of imaginary to real part of the measurement signal.
